# Supplementary figures and images for: sumSTAAR: A flexible framework for gene-based association studies using GWAS summary statistics
Source: PLoS Comput Biol. 2022 Jun 2;18(6):e1010172. doi: 10.1371/journal.pcbi.1010172 (PMC9197066; doi:10.1371/journal.pcbi.1010172)

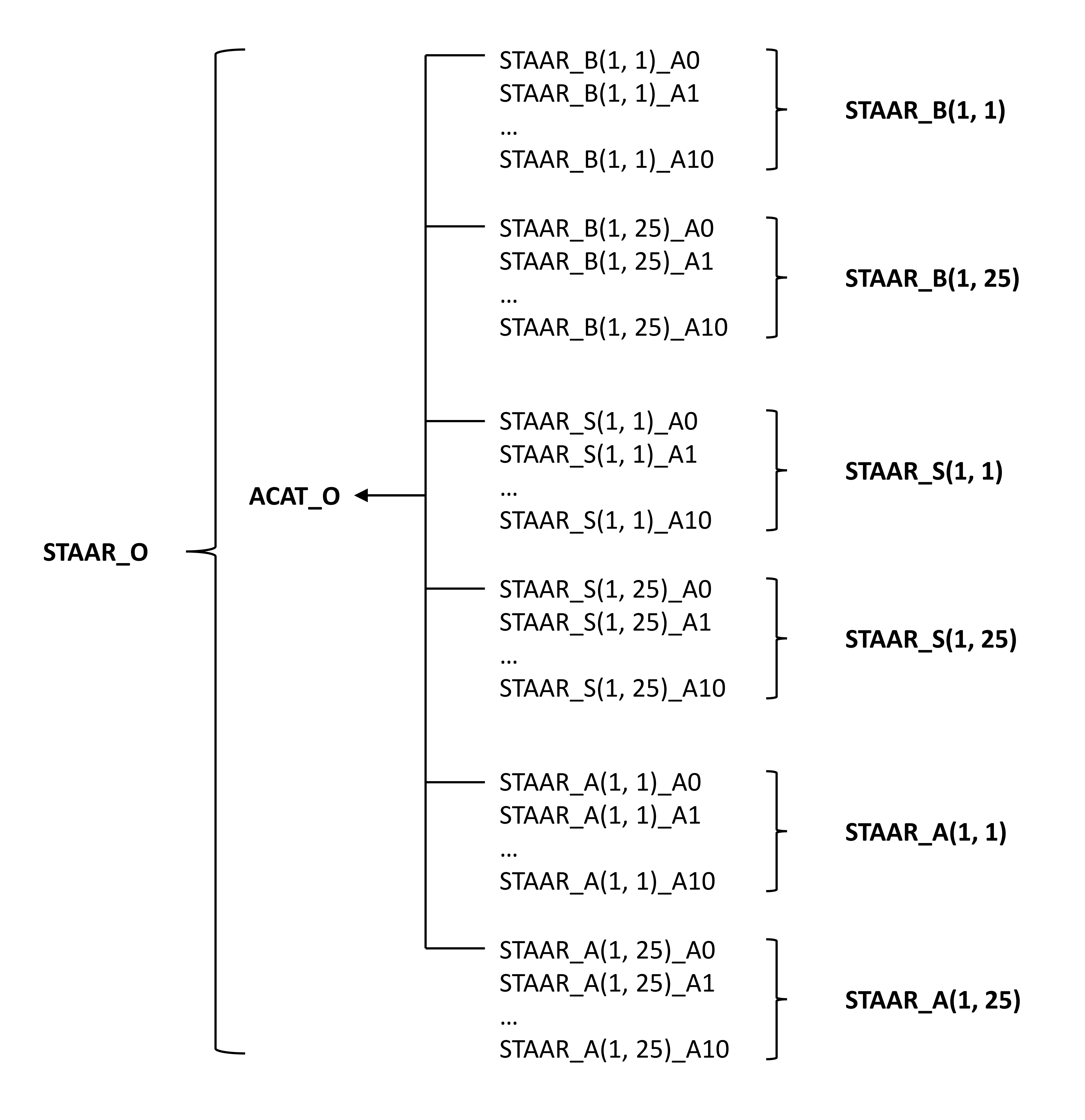

Supplement: S1 Fig — Combined tests are shown in bold. (TIF) [file pcbi.1010172.s001.tif]

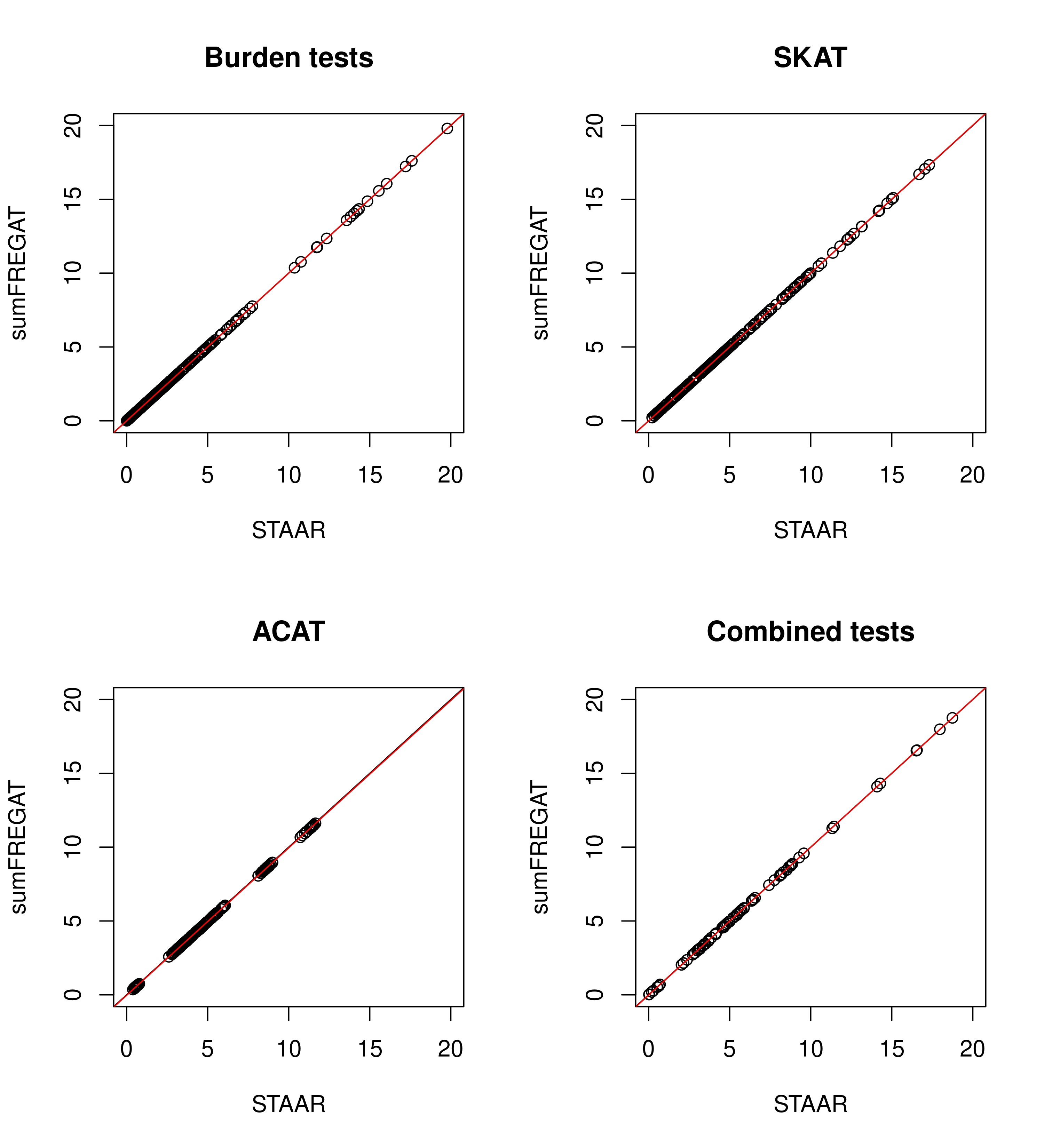

Supplement: S2 Fig — Negative log10(p-value) were calculated in 10 simulations. The first three panels show the results for individual gene-based tests (Burden test, SKAT and ACAT) with two sets of parameters for the Beta distribution and 11 variants of annotation weighting. The last panel presents–log10(p-values) for all combined tests. The regression lines are shown in red (overlap the lines of one-to-one correspondence). (TIF) [file pcbi.1010172.s002.tif]

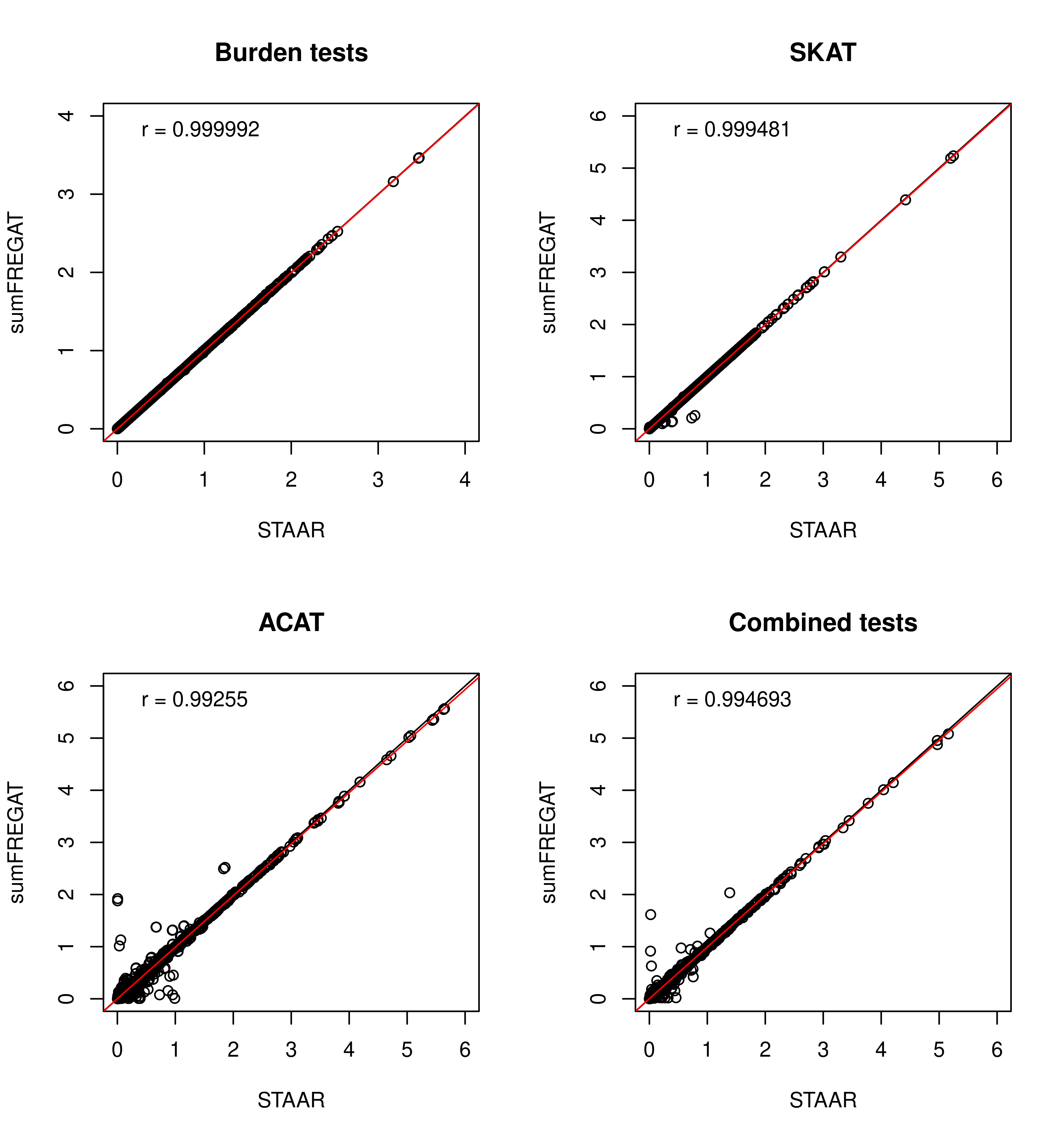

Supplement: S3 Fig — The -log10 transformed p-value of each gene is shown. The first three panels show the results for individual gene-based tests (Burden test, SKAT and ACAT) with two sets of parameters for the Beta distribution. The last panel presents results combined across all tests. The regression lines are shown in red (overlap the black lines of one-to-one correspondence); ‘r’ is the correlation coefficient. (TIF) [file pcbi.1010172.s003.tif]
