# Supplementary material for: sumSTAAR: A flexible framework for gene-based association studies using GWAS summary statistics
Source: PLoS Comput Biol. 2022 Jun 2;18(6):e1010172. doi: 10.1371/journal.pcbi.1010172 (PMC9197066; doi:10.1371/journal.pcbi.1010172)
Supplement: S1 Text — (DOCX) [file pcbi.1010172.s004.docx]

**Introducing the probabilities of genetic variants being causal in the different methods**

A great number of summary SNP-level statistics such as z-scores (*z*), p-values (*p*) and effect sizes (*β*) are now available for different traits and diseases in open-access databases [1]. By definition, for each genetic variant, z-score follows the standard normal distribution *N*(0,1) under the null hypothesis of no association between the variant and the trait, and can be easily calculated from another GWAS summary statistics, namely the p-value and *β*: the absolute value of a z-score is defined by the p-value as: |*z*| = Φ^-1^(1-*p*/2), where Φ is a standard normal cumulative distribution function, and the z-score sign is defined by the sign of the corresponding *β*.

For a sample of unrelated individuals, a vector of z-scores, *Z*, obtained one by one for each of *M* SNPs in the gene is defined as:

$$Z=\frac{V^{-1}\tilde{G}^{T}\tilde{y}}{\sqrt{N}\sigma_{y}}.$$

Here $\tilde{G}$ and $\tilde{y}$ denote the centered values of an (*N×M*) matrix of genotypes and an (*N×*1) vector of phenotypes, respectively; *V* is an (*M×M*) diagonal matrix of the square roots of genotypic variances defined via $\sqrt{se\left( \beta\right)}$ or $\sqrt{MAF\left( 1-MAF \right)}$ under Hardy-Weinberg equilibrium, where *se*() denotes standard errors, and *MAF* is minor allelic frequencies; *N* is the sample size and *σ_y_* is the phenotypic variance.

For *M* genetic variants, *Z* follows the multivariate normal distribution *N*(0,*U*) under the null hypothesis of no association between the genetic variants and the trait, where *U* is calculated as an (*M*×*M*) matrix of correlations between the genotypes of these variants [2]:

$$U=\frac{V^{-1}\tilde{G}^{T}\tilde{G}V^{-1}}{N}.$$

The *U* matrix can be estimated using a reference sample of genotypes originating from the same population.

In the methods implemented in sumFREGAT and using the multiple linear regression models, the test statistics and their distributions are presented in terms of *Z* and *U* that can be weighted in a certain way.

For Burden test (BT) and SKAT based on the random effects models, test statistics are:

$$\begin{matrix} Q_{BT,i,j}=\left( Z^{T}VW_{i}{}_{j}e \right)^{2}, \\ Q_{SKAT,i,j}=Z^{T}VW_{i}{}_{j}W_{i}VZ, \end{matrix} (1)$$

where *W_i_* is a diagonal matrix of SNP weights defined via the Beta density function of MAFs with the *i*-th set of parameters; *Π_j_* is a diagonal matrix of the probabilities of genetic variants being causal defined by the *j*-th functional annotation, and *e* is the vector of units.

Under the null hypothesis, *Q_BT_* follows a scaled χ^2^ distribution with one degree of freedom, $\lambda\chi_{\mathrm{df}=1}^{2}$, where *λ* $=e^{T}{}_{j}W_{i}VUVW_{i}{}_{j}e$, and *Q_SKAT_* follows a weighted sum of χ^2^_df=1_ distributions, $\sum\lambda\chi_{\mathrm{df}=1}^{2}$, where *λ* are eigenvalues of $W_{i}VUVW_{i}{}_{j}$*.*

Another kernel-based method presented in sumFREGAT is SKAT-O [3-5]. Since this method is a linear combination of BT and SKAT, *Q_SKATO_* follows the weighted sum of χ^2^_df=1_ distributions, where weights are the eigenvalues of the kernel matrix*.* For SKAT-O, the probabilities of genetic variants being causal (*Π_j_*) can be introduced into test statistics and eigenvalues of the kernel matrix as follows:

$$\begin{matrix} Q_{SKATO,i,j}=\left( Z^{T}VW_{i}{}_{j}e \right)^{2}+\left( 1- \right)Z^{T}VW_{i}{}_{j}W_{i}VZ \\ =eigen\left( \left( U^{\frac{1}{2}}VW_{i}{}_{j}ee^{T}{}_{j}W_{i}VU^{\frac{1}{2}} \right)+\left( 1- \right)\left( U^{\frac{1}{2}}VW_{i}{}_{j}W_{i}VU^{\frac{1}{2}} \right) \right). \end{matrix}$$

where *ρ* is the coefficient of the linear combination of tests.

The methods based on multiple linear regression models with fixed genetic effects use the F-test statistic to test the null hypothesis H_0_: *β* = 0 against H_1_: *β*≠ 0. For the complete multiple linear regression model (MLR), the F-test statistic for a region with *M* genetic variants is calculated by [6]:

$$F=\frac{R^{2}\left( N-M-1 \right)}{\left( 1-R^{2} \right)M},$$

where

$$R^{2}=\frac{Z^{T}U^{-1}Z}{N}.$$

The p-value for MLR is defined by an F-distribution with *M* and *N-M-*1 as the numbers of degrees of freedom. As can be seen, MLR requires the inverse of the correlation matrix *U*. If the columns of *U* are collinear or nearly collinear, numerical instability and potentially inflated regression coefficients may appear. On average, collinearity in *U* substantially depends on SNP density, i.e. the number of SNPs analyzed. New dense correlation matrices proposed in this study provide 4.65 times higher SNP coverage and, therefore, higher collinearity in *U*. Taking into account the sensitivity of MLR to the increased collinearity, we did not include MLR in our updated version of sumFREGAT.

In contrast to MLR, the PCA [7] and FLM [8-10] methods use incomplete (truncated) linear regression models with fixed effects. Both methods serve to reduce the number of regression predictors by generating an orthogonal basis set of *K* elements. For PCA, these elements are the first *K* principal components containing a large proportion of information about genotype data. For FLM, these elements are the *K* basis functions, by which the genotypes and their effects can be presented as continuous functions. For incomplete regression models, *R*^2^ is calculated as

$$R^{2}=\frac{Z^{T}{VW_{i}C\left( C^{T}W_{i}VUVW_{i}C \right)}^{-1}C^{T}W_{i}VZ}{N}, \left( 4 \right)$$

where *C* is the (*M*×*K*) matrix specified for each gene-based method [9, 10]. For FLM, each element of *C*, *C_mk_*, represents the value of the *k*-th basis function calculated at the *m*-th genetic variant position. For PCA, *C* is given as a truncated matrix of right singular vectors obtained from the singular value decomposition of the weighted genotype matrix. Truncating is achieved by considering only the first *K* largest squared singular values that account for 80-90% of the total genotype variance observed in the genomic region [7, 11]. For methods using incomplete multiple linear regression models, the value of *M* in the F-test numbers of degrees of freedom is replaced by

$$M'=rank\left( C^{T}W_{i}VUVW_{i}C \right).$$

The probabilities of genetic variants being causal (*Π_j_*) are introduced in Expression (4) as follows:

$$R^{2}=\frac{Z^{T}{VW_{i}{}_{j}C\left( C^{T}{}_{j}W_{i}VUVW_{i}{}_{j}C \right)}^{-1}C^{T}{}_{j}W_{i}VZ}{N}$$

and

$$M'=rank\left( C^{T}{}_{j}W_{i}VUVW_{i}{}_{j}C \right).$$

PCA is a robust method, because multicollinearity is completely eliminated by orthogonal conversion of correlated predictors to principal components (PCs) that are linearly uncorrelated. PCA always ensures *Mʹ* = *K*.

In contrast, the FLM results are not always stable because FLM minimizes but does not eliminate multicollinearity. The loss of orthogonality still may occur in the matrices of basis functions calculated for some genes. To guard against this problem, we introduced a filter to ensure that matrix to be inverted in FLM is full rank (*Mʹ* = *K*). Due to this filter, FLM is applied to a restricted set of genes.

One more method introduced in sumFREGAT is an aggregated Cauchy association test (ACAT-V) proposed by Liu, et al., 2019 [12] and modified by Li, et al., 2020 [13]. The gene-based analysis test is defined as:

$$T_{ACAT-V_{i,j}}=\frac{1}{W_{sum}}{[_{j}}_{0}{w_{i}}_{0}^{2}\sigma_{g_{0}}^{2}]\tan\left\{ \left( 0.5-p_{0} \right)\pi\right\}+$$

$$\frac{1}{W_{sum}}\sum_{m=M_{0}+1}^{M} {{}_{j}}_{m}{w_{i}}_{m}^{2}\sigma_{g_{m}}^{2}\tan\left\{ \left( 0.5-p_{m} \right)\pi\right\},$$

where the first summand is applied to the *M*_0_ extremely rare variants with MAC ≤ 10, and the second to the rest variants. Here ${{}_{j}}_{m}$ is the probability of *m*-th variant being causal defined by the *j*-th functional annotation; ${w_{i}}_{m}$ is the weight of *m*-th variant defined by the Beta density function of MAF with the *i*-th set of parameters; $\sigma_{g_{m}}^{2}$ is introduced to make the total weights in ACAT-V comparable with those in methods using linear regression models, $\sigma_{g_{m}}\sim V_{mm}$. In the first summand, ${[_{j}}_{0}{w_{i}}_{0}^{2}\sigma_{g_{0}}^{2}]$ is the average of the weights among the extremely rare variants, *p*_0_ is the Burden test p-value calculated for these variants. $W_{sum}={[_{j}}_{0}{w_{i}}_{0}^{2}\sigma_{g_{0}}^{2}]+\sum_{m=1}^{M} {{}_{j}}_{m}{w_{i}}_{m}^{2}\sigma_{g_{m}}^{2}$.

To combine *L* different methods, ACAT is used without any weighting:

$$T_{ACAT}=\frac{1}{L}\sum_{l=1}^{L} \tan\left\{ \left( 0.5-p_{l} \right)\pi\right\}.$$

The p-values for ACAT-V and ACAT tests are calculated as:

$$p_{ACAT}= 0.5-\frac{arctg\left( T_{ACAT} \right)}{\pi}.$$

**Equivalence of methods implemented in the sumFREGAT and STAAR packages**

The formulas for gene-based association test statistics proposed by Li, et al., 2020 [13] are written in terms of *s* but not *z*-score statistics. It is easy to see that

$$S=VZ. (2)$$

Formula (2) follows from the equality $G^{T}\tilde{y}=\tilde{G}^{T}\tilde{y}$ explained by the idempotent property of the centering projection *P* matrix: *PP=P*, where *P*= *I_N_* – *X*(*X^T^X*)^-1^*X^T^*, and *X* is the matrix of covariates taking into account the intercept. Since $\tilde{y}$ can be expressed as $\tilde{y}=Py$,

$G^{T}\tilde{y}=G^{T}Py=G^{T}PPy=\tilde{G}^{T}\tilde{y}$.

To rewrite the formulas for gene-based association tests proposed by Li, et al., 2020 [13] in terms of z-scores, we present these formulas in matrix form:

$$\begin{matrix} Q_{Burden,i,j}=\left( \sum_{m=1}^{M} {}_{jm}w_{im}S_{m} \right)^{2}=S^{T}W_{i}\left( {}_{j}ee^{T}{}_{j} \right)W_{i}S, \\ Q_{SKAT,i,j}=\sum_{m=1}^{M} {}_{jm}w_{im}^{2}S_{m}^{2}=S^{T}W_{i}\left( {}_{j} \right)W_{i}S. \end{matrix} (3)$$

Substituting Expression (2) into Expressions (3) gives

$Q_{BT,i,j}={(Z^{T}VW_{i}{}_{j}e)}^{2}$,

$Q_{SKAT,i,j}=Z^{T}VW_{i}{}_{j}W_{i}VZ$.

Therefore, Formulas (3) proposed by Li, et al., 2020 [13] can be presented in terms of summary z-score statistics and the *U* matrix*.* The obtained formulas are equivalent to Formulas (1) implemented in sumFREGAT.

**References**

1. Pasaniuc B, Price AL. Dissecting the genetics of complex traits using summary association statistics. Nature reviews Genetics. 2017;18(2):117-27. doi: 10.1038/nrg.2016.142. PubMed PMID: 27840428; PubMed Central PMCID: PMCPMC5449190.

2. Conneely KN, Boehnke M. So many correlated tests, so little time! Rapid adjustment of P values for multiple correlated tests. Am J Hum Genet. 2007;81(6):1158-68. doi: 10.1086/522036. PubMed PMID: 17966093; PubMed Central PMCID: PMCPMC2276357.

3. Svishcheva GR. A generalized model for combining dependent SNP-level summary statistics and its extensions to statistics of other levels. Sci Rep. 2019;9(1):5461. doi: 10.1038/s41598-019-41827-5. PubMed PMID: 30940856; PubMed Central PMCID: PMCPMC6445108.

4. Svishcheva GR, Belonogova NM, Zorkoltseva IV, Kirichenko AV, Axenovich TI. Gene-based association tests using GWAS summary statistics. Bioinformatics. 2019;35(19):3701-8.

5. Lee S, Wu MC, Lin X. Optimal tests for rare variant effects in sequencing association studies. Biostatistics. 2012;13(4):762-75. doi: 10.1093/biostatistics/kxs014. PubMed PMID: 22699862; PubMed Central PMCID: PMC3440237.

6. Chow GC. Tests of Equality Between Sets of Coefficients in Two Linear Regressions. Econometrica. 1960;28(3):591-605.

7. Wang K, Abbott D. A principal components regression approach to multilocus genetic association studies. Genet Epidemiol. 2008;32(2):108-18. doi: 10.1002/gepi.20266. PubMed PMID: 17849491.

8. Fan R, Wang Y, Mills JL, Wilson AF, Bailey-Wilson JE, Xiong M. Functional linear models for association analysis of quantitative traits. Genet Epidemiol. 2013;37(7):726-42. doi: 10.1002/gepi.21757. PubMed PMID: 24130119; PubMed Central PMCID: PMCPMC4163942.

9. Svishcheva GR, Belonogova NM, Axenovich TI. Region-Based Association Test for Familial Data under Functional Linear Models. PLoS One. 2015;10(6):e0128999. doi: 10.1371/journal.pone.0128999. PubMed PMID: 26111046; PubMed Central PMCID: PMCPMC4481467.

10. Belonogova NM, Svishcheva GR, Wilson JF, Campbell H, Axenovich TI. Weighted functional linear regression models for gene-based association analysis. PLoS One. 2018;13(1):e0190486. doi: 10.1371/journal.pone.0190486. PubMed PMID: 29309409; PubMed Central PMCID: PMCPMC5757938.

11. Gauderman WJ, Murcray C, Gilliland F, Conti DV. Testing association between disease and multiple SNPs in a candidate gene. Genet Epidemiol. 2007;31(5):383-95. doi: 10.1002/gepi.20219. PubMed PMID: 17410554.

12. Liu Y, Chen S, Li Z, Morrison AC, Boerwinkle E, Lin X. ACAT: A Fast and Powerful p Value Combination Method for Rare-Variant Analysis in Sequencing Studies. Am J Hum Genet. 2019;104(3):410-21. doi: 10.1016/j.ajhg.2019.01.002. PubMed PMID: 30849328; PubMed Central PMCID: PMCPMC6407498.

13. Li X, Li Z, Zhou H, Gaynor SM, Liu Y, Chen H, et al. Dynamic incorporation of multiple in silico functional annotations empowers rare variant association analysis of large whole-genome sequencing studies at scale. Nat Genet. 2020;52(9):969-83. doi: 10.1038/s41588-020-0676-4. PubMed PMID: 32839606; PubMed Central PMCID: PMCPMC7483769.
