# Supplementary material for: sumSTAAR: A flexible framework for gene-based association studies using GWAS summary statistics
Source: PLoS Comput Biol. 2022 Jun 2;18(6):e1010172. doi: 10.1371/journal.pcbi.1010172 (PMC9197066; doi:10.1371/journal.pcbi.1010172)
Supplement: S2 Text — (DOCX) [file pcbi.1010172.s005.docx]

**Comparison of STAAR and sumSTAAR using simulated data**

We tested the equivalence of the results obtained by STAAR and sumFREGAT. The STAAR procedure suggests using a combination of three gene-based tests (Burden test, SKAT and ACAT-V) and two sets of Beta distribution parameters: (1, 1) and (1, 25). For each test and each set of parameters, STAAR calculates 11 individual tests: the original test and 10 tests weighted for each of 10 annotations. The resulting values are combined using the Cauchy method into six STAAR tests (see Fig. S1). P-values from all original (A0) tests are also combined into the ACAT_O p-value. All individual test p-values are combined into the STAAR_O p-value.

Using summary statistics, we reproduced this procedure in sumFREGAT. With the Example data and code from STAAR, we simulated phenotypes and the probabilities of variants being causal for 10,000 individuals and a 5,000-bp region randomly selected on a chromosome 1,000,000 bp in size. The number of SNPs in the region typically fell in the range from 120 to 170, all SNPs having MAF ≤ 0.05. With the individual genotype and phenotype data, we performed all individual and combined tests using the original STAAR procedure. We also calculated z-scores and effect sizes for each variant and the matrix of genotype correlations *U*.


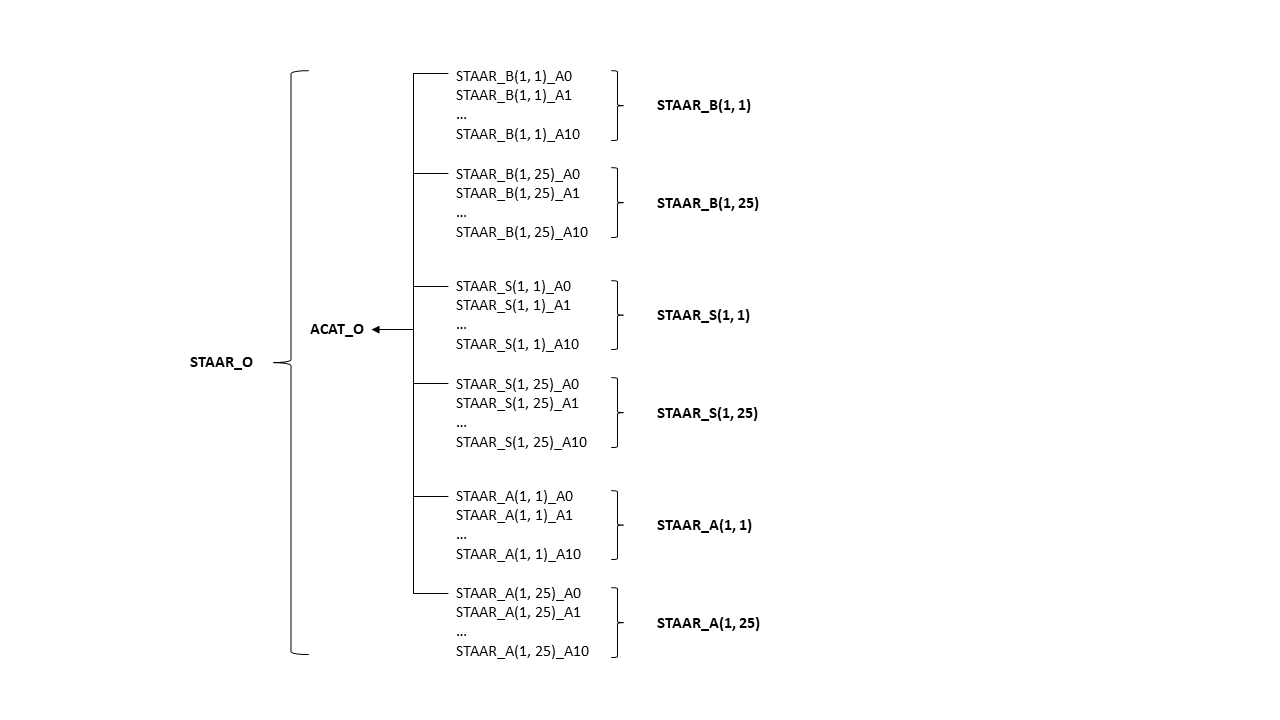


**Fig. S1. Tests performed within the STAAR procedure.** Combined tests are shown in bold.

Next, we used these data as inputs in our sumSTAAR() function of the sumFREGAT package to obtain the same p-values on the summary statistics. R code to perform simulations and comparisons is available at https://github.com/nbelon/sumSTAAR-vs-STAAR-comparison/blob/main/sumSTAAR.vs.STAAR.R. Example data used for simulations contain genotypes of 30,095 for 10,000 individuals, MACs ranging from 1 to 999 with median 3. As can be seen in Fig. S2, there is excellent agreement between the results obtained by two packages.


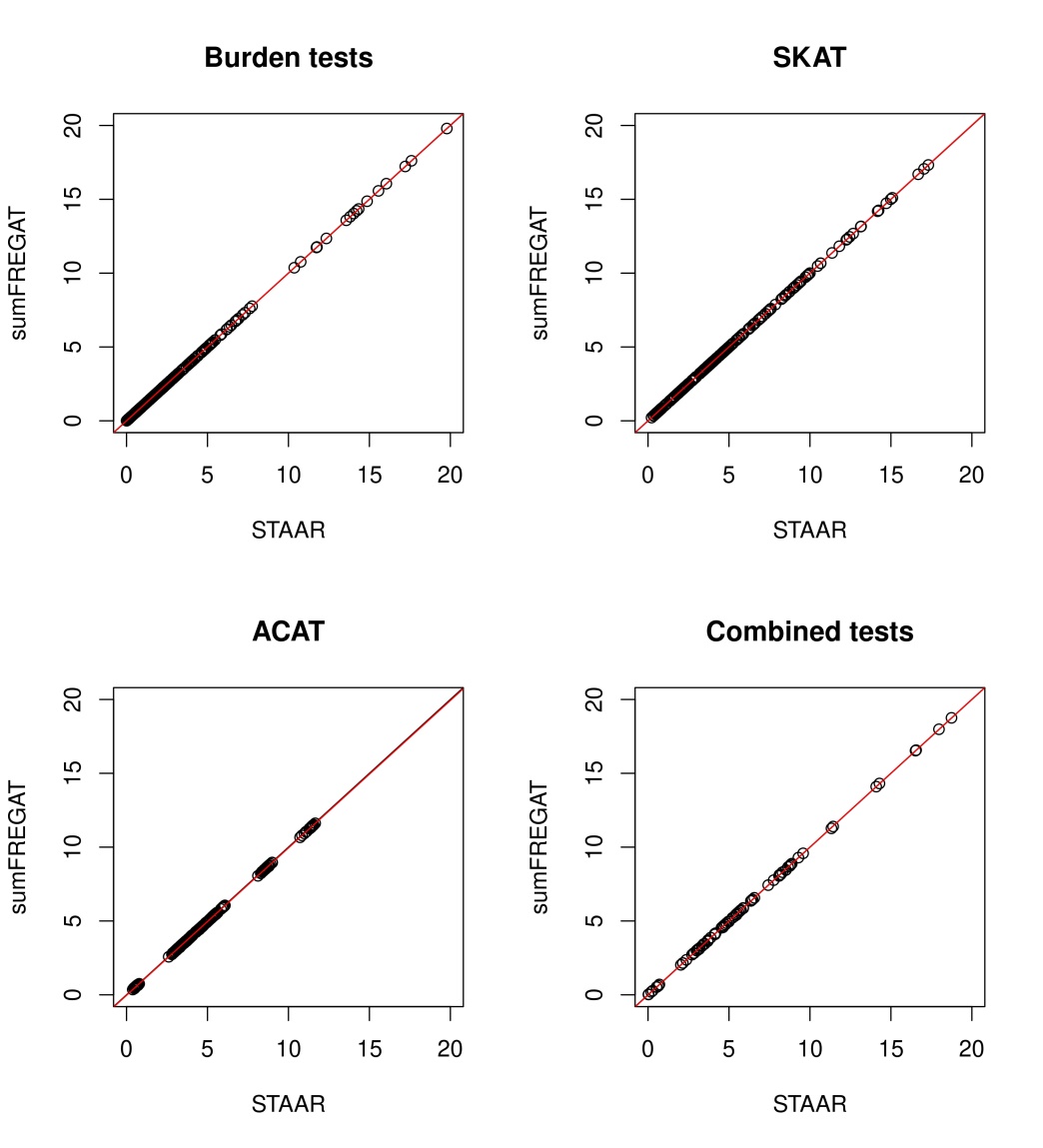


**Fig. S2**. **Comparison of the results obtained by the STAAR and sumFREGAT packages**. Negative log_10_(p-value) were calculated in 10 simulations. The first three panels show the results for individual gene-based tests (Burden test, SKAT and ACAT) with two sets of parameters for the Beta distribution and 11 variants of annotation weighting. The last panel presents –log_10_(p-values) for all combined tests. The regression lines are shown in red (overlap the lines of one-to-one correspondence).
