# Supplementary material for: sumSTAAR: A flexible framework for gene-based association studies using GWAS summary statistics
Source: PLoS Comput Biol. 2022 Jun 2;18(6):e1010172. doi: 10.1371/journal.pcbi.1010172 (PMC9197066; doi:10.1371/journal.pcbi.1010172)
Supplement: S3 Text — (DOCX) [file pcbi.1010172.s006.docx]

**Comparison of STAAR and sumSTAAR using real exome sequencing data**

We used UK Biobank whole exome sequencing data and phenotype of the chronic ischaemic heart disease (ICD-10 code I25) for 153,379 unrelated individuals with European ancestry (project #59345). We analyzed 105,310 variants covering 1,927 genes from chromosome 1 after the following filters: call rate = 1, MAC ≥ 5 and MAF < 0.01. The variants were annotated to the genes based on dbSNP version 143 SNP locations and the GRCh38.p13 genome assembly. SNPs were annotated to a gene if they were located between its transcription start and stop sites. Sex, age and batch were used as covariates of the phenotype (12,931 cases / 140,448 controls).

Using the original STAAR procedure and the individual genotype and phenotype data, we performed three gene-based tests (Burden test, SKAT and ACAT-V) each with two sets of Beta distribution parameters, (1, 1) and (1, 25), and their combination.

The same genotype and phenotype data were used for calculating the summary statistics and LD correlations. The summary statistics were obtained using fastGWA-GLMM tool [1]. The z-scores were calculated as T/SE_T. LD matrices were calculated for 18,386 genes using exome sequences of 153,383 participants of European ancestry and LDstore software v.2.0 [2]. Only variants with MAC ≥ 3 were used. In total, matrices contain correlations for 5,225,822 SNPs, with 5,063,068 (97%) of them having MAF < 0.01. These LD matrices calculated on exome sequencing data are freely available at https://mga.bionet.nsc.ru/ukbb_exome_matrix/.

We verified that the p-values obtained by the STAAR and fastGWA-GLMM packages are very close:


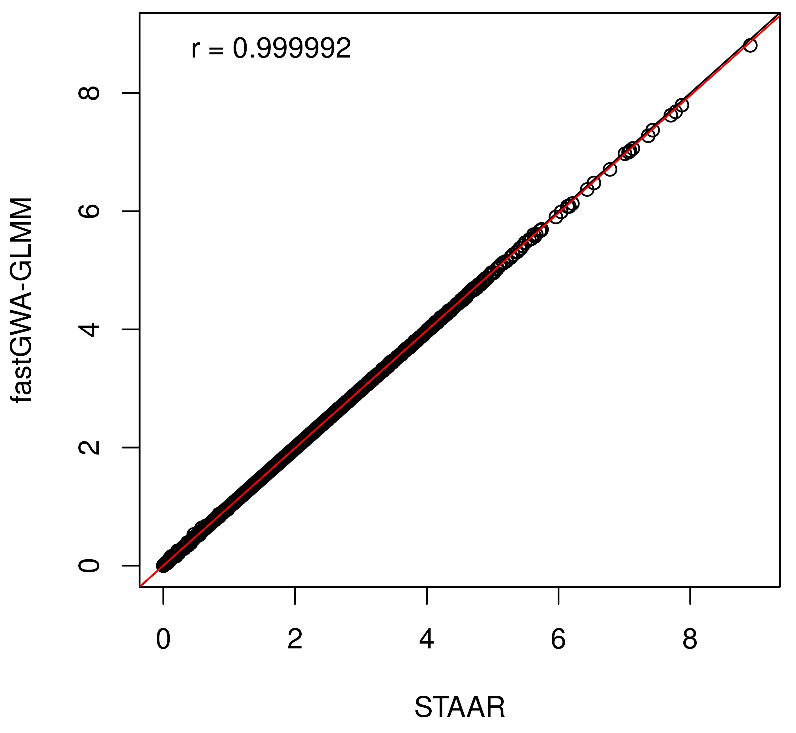


**Comparison of variant p-values obtained by the STAAR and** **fastGWA-GLMM packages**. The –log10 transformed p-value of each variant is shown. The regression line is shown in red (overlaps the black line of one-to-one correspondence); ‘r’ is the correlation coefficient.

Using the summary statistics and LD correlations, we reproduced the gene-based analyses by the sumSTAAR() function of the sumFREGAT package. As can be seen in Fig. S3, there is a good agreement between the results obtained by the two packages. Observed discrepancies probably result from small differences in summary statistics. In particular, ACAT is very sensitive to little changes in the p-values at both edges of the distribution, including those close to 1. High precision for large p-values is not a priority for GWAS tools, and correlation coefficient between –log10(1–p) for individual variant p-values obtained by STAAR and fastGWA-GLMM was 0.9986 instead of 1 for –log10(p). Correlation coefficient for ACAT increased from 0.993 to 0.999 when using the individual p-values produced by STAAR instead of fastGWA-GLMM. Methods using LD matrices (BT, SKAT) show rather good correspondence even with fastGWA-GLMM summary statistics.


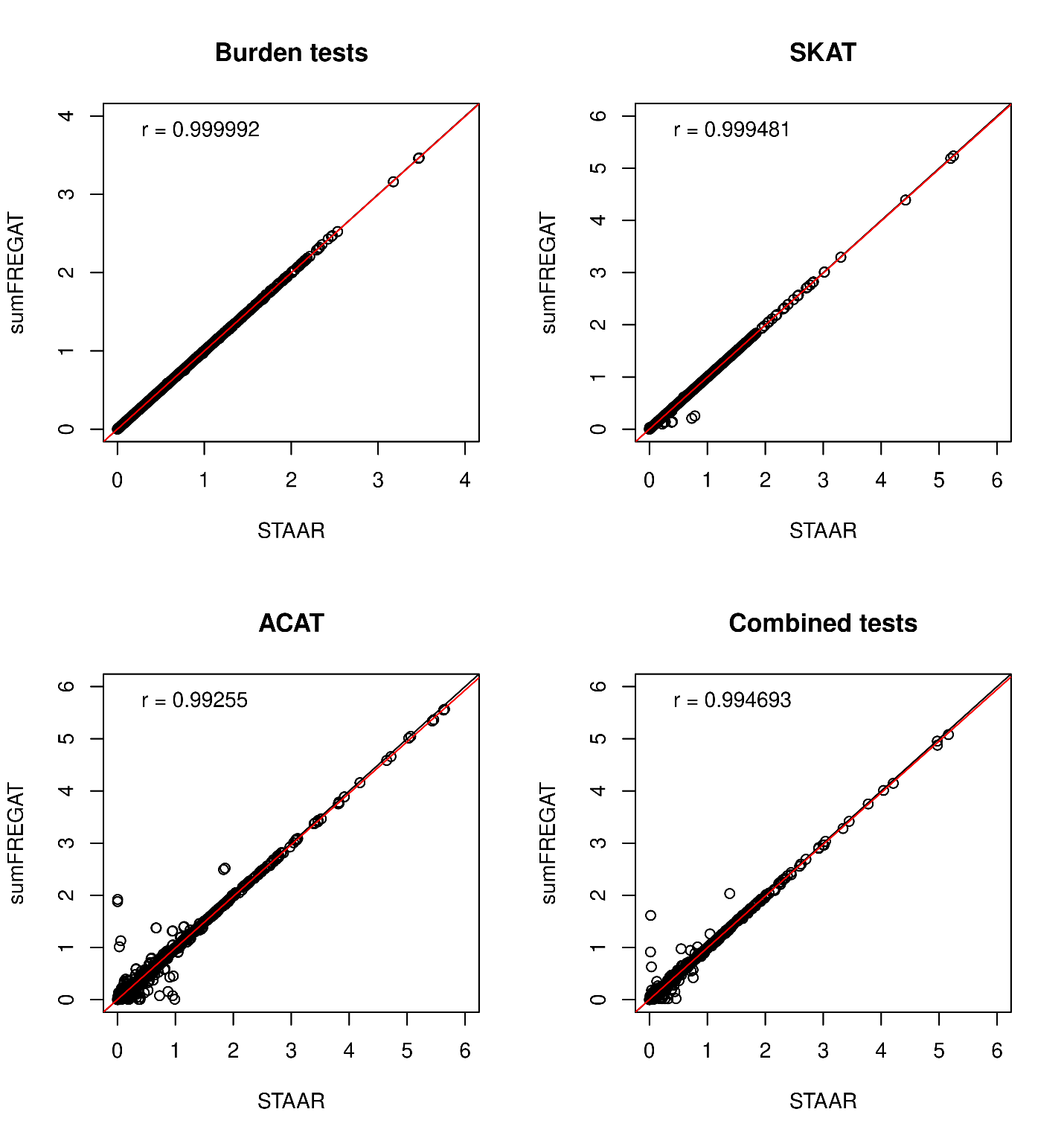


**Fig. S3**. **Comparison of the results obtained by the STAAR and sumFREGAT packages**.

The –log10 transformed p-value of each gene is shown. The first three panels show the results for individual gene-based tests (Burden test, SKAT and ACAT) with two sets of parameters for the Beta distribution. The last panel presents results combined across all tests. The regression lines are shown in red (overlap the black lines of one-to-one correspondence); ‘r’ is the correlation coefficient.

The Q-Q plots for STAAR and sumSTAAR are almost identical, with sumSTAAR being slightly more conservative (Fig. S4).


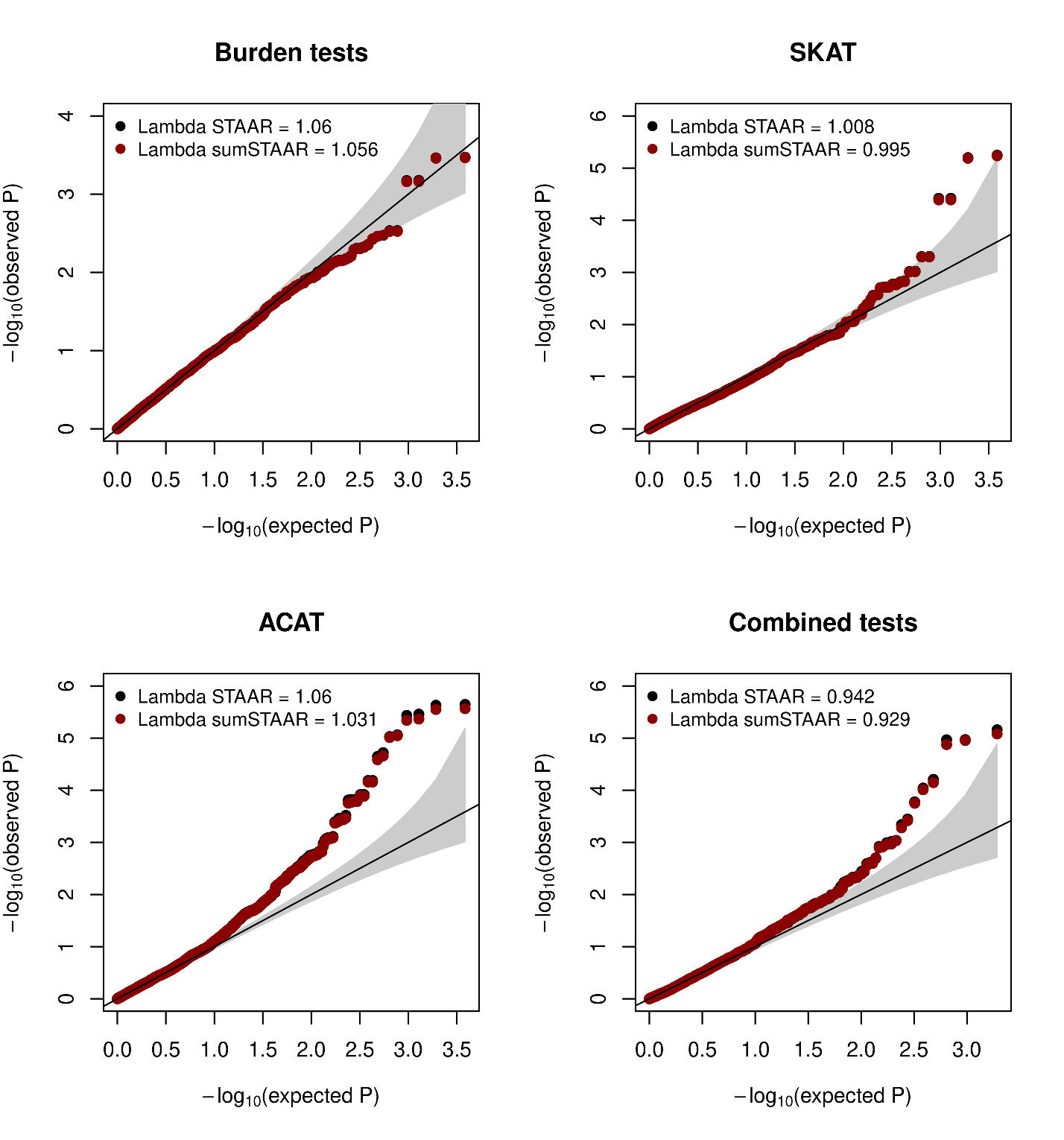


**Fig. S4**. **Comparison of the Q-Q plots for the STAAR and sumFREGAT tests**.

The same tests as in Fig. S3 are shown. The black line is the regression line. The 95% confidence interval is shown in grey.

References

1. Jiang L, Zheng Z, Fang H, Yang J. A generalized linear mixed model association tool for biobank-scale data. Nat Genet. 2021;53(11):1616-21. doi: 10.1038/s41588-021-00954-4. PubMed PMID: 34737426.

2. Benner C, Havulinna AS, Järvelin M-R, Salomaa V, Ripatti S, Pirinen M. Prospects of fine-mapping trait-associated genomic regions by using summary statistics from genome-wide association studies. The American Journal of Human Genetics. 2017;101(4):539-51.
