# Supplementary material for: sumSTAAR: A flexible framework for gene-based association studies using GWAS summary statistics
Source: PLoS Comput Biol. 2022 Jun 2;18(6):e1010172. doi: 10.1371/journal.pcbi.1010172 (PMC9197066; doi:10.1371/journal.pcbi.1010172)
Supplement: S4 Text — (DOCX) [file pcbi.1010172.s007.docx]

**SumSTAAR procedure in application to real data**

We used the open-access neuroticism summary statistics calculated from imputed genotypes (described in the main text) to assess the effectiveness of introduction of functional annotations, different methods and weighting functions within the sumSTAAR procedure. Genotypes of the 1000 Genome Genomes Project were used to perform the polygene pruning that excluded all SNPs having *r*^2^ > 0.2 with genome-wide significant SNPs (p < 2.5 × 10^-8^) located outside the regions of interest. We analyzed only exonic SNPs that passed polygene pruning (98,964 SNPs assigned to 13,100 genes).

We used nine annotation scenarios: eight with integrative scores (aPCs) from FAVOR v.2 (http://favor.genohub.org/) [1] and a scenario without annotations. We also used two standard schemes of weighting by MAF: the Beta density function of MAFs with parameters (1, 1) or (1, 25). In total, 72 tests were performed with four gene-based methods (BT, SKAT, PCA and ACAT-V), two weighting functions, and nine annotation scenarios. The obtained p-values were then combined by gene-based methods, weighting functions, and whether functional annotations were used or not. This resulted in 8 combined tests, all calculated by ACAT. Table S1 represents the resulting p-values for 10 genes showing p < 2.5 × 10^-5^ for at least one of the 8 combined tests.

As can be seen from Table S1, the use of functional annotations reduced the p-values for 8 out of 10 genes. Four gene-based methods favored different genes, with each method having at least two best p-values when compared with other three methods. The MAF-unweighted scheme with parameters (1, 1) proved to be beneficial in this analysis, possibly due to the lack of extremely rare variants. However, one gene, *NARF*, was identified due to both the Beta distribution parameters (1, 25) and functional annotations. To conclude, functional annotations, wider range of methods and weighting functions within the sumSTAAR framework provides an optimal testing procedure with benefits of different settings combined together.

**Table S1**. **The results of gene-based association analysis combining different tests**

| **Gene** | **Annotations^a^** | |  | **Methods^b^** | | | |  | **Weighting functions^c^** | |
| --- | --- | --- | --- | --- | --- | --- | --- | --- | --- | --- |
|  | + | - |  | PCA | SKAT | BT | ACAT-V |  | (1, 1) | (1, 25) |
| *ME3* | **4.09E-06** | **5.51E-06** |  | **4.41E-06** | **2.02E-06** | 8.79E-01 | **3.47E-06** |  | **1.98E-06** | 8.13E-01 |
| *NOS1* | **1.58E-08** | **1.62E-08** |  | **7.24E-08** | **7.85E-09** | **2.72E-06** | **8.87E-09** |  | **7.87E-09** | 1.26E-04 |
| *CSNK1G1* | **3.94E-06** | **4.12E-06** |  | **1.45E-06** | 2.85E-03 | 2.29E-03 | **3.02E-06** |  | **2.53E-06** | **8.71E-06** |
| *AGBL1* | **7.34E-07** | **1.02E-06** |  | **2.30E-07** | 6.40E-05 | 3.54E-04 | **7.88E-07** |  | **3.54E-07** | 1.11E-01 |
| *FBXL19* | **1.50E-05** | **1.50E-05** |  | **1.87E-05** | **1.98E-05** | **8.63E-06** | **2.11E-05** |  | **1.47E-05** | **1.52E-05** |
| *NARF* | **2.48E-05** | 2.69E-05 |  | 2.62E-05 | **1.42E-05** | 2.87E-03 | **1.87E-05** |  | 1.31E-04 | **1.36E-05** |
| *PHAX* | **8.61E-06** | **6.89E-06** |  | 6.62E-05 | 6.68E-05 | **2.40E-06** | 3.50E-04 |  | **6.54E-06** | **1.39E-05** |
| *TRIM39-RPP21* | **1.43E-08** | **1.64E-08** |  | **1.95E-08** | **9.97E-08** | **4.09E-06** | **4.49E-09** |  | **7.04E-09** | **2.30E-05** |
| *RPP21* | **1.35E-08** | **1.61E-08** |  | **2.30E-08** | **1.42E-07** | **2.39E-05** | **3.99E-09** |  | **6.65E-09** | **1.61E-05** |
| *SLC25A37* | **8.03E-06** | **1.26E-05** |  | **9.26E-06** | **9.76E-06** | 2.03E-01 | **3.22E-06** |  | **3.84E-06** | 1.28E-01 |

P-values < 2.5 × 10^-5^ shown in bold.

^a^ +: Functional annotations used (analogous to the STAAR-O p-value of STAAR). The number of tests combined was 72 = 4 methods x (8+1) annotation scenarios x 2 weighting functions

–: No functional annotations used (analogous to the ACAT-O p-value of STAAR). The number of tests combined was 8 = 4 methods × 2 weighting functions.

Lower p-value in a pair shown in green.

^b^ for each method, 18 tests ((8+1) annotation scenarios x 2 weighting functions) were combined. Lowest p-value among four methods shown in green.

^c^ for each weighting function, 36 tests (4 methods x (8+1) annotation scenarios) were combined. Lower p-value in a pair shown in green.

In the gene-based analysis of neuroticism, the inflation coefficients (lambdas) proved to be higher than in the chronic ischaemic heart disease analysis described in S3 Text (Fig. S5 below vs Fig. S4 in S3 Text). A possible explanation is the higher statistical power of neuroticism study: 380,506 individuals instead of 153,379 (~2.5 times difference), common alleles along with rare ones. Nevertheless, the inflation coefficient of the total combined test (first panel in the Fig. S5) was notably lower than that for most individual gene-based tests (SKAT, PCA, and ACAT-V). Yet, the combined test retained the top hits of individual tests.


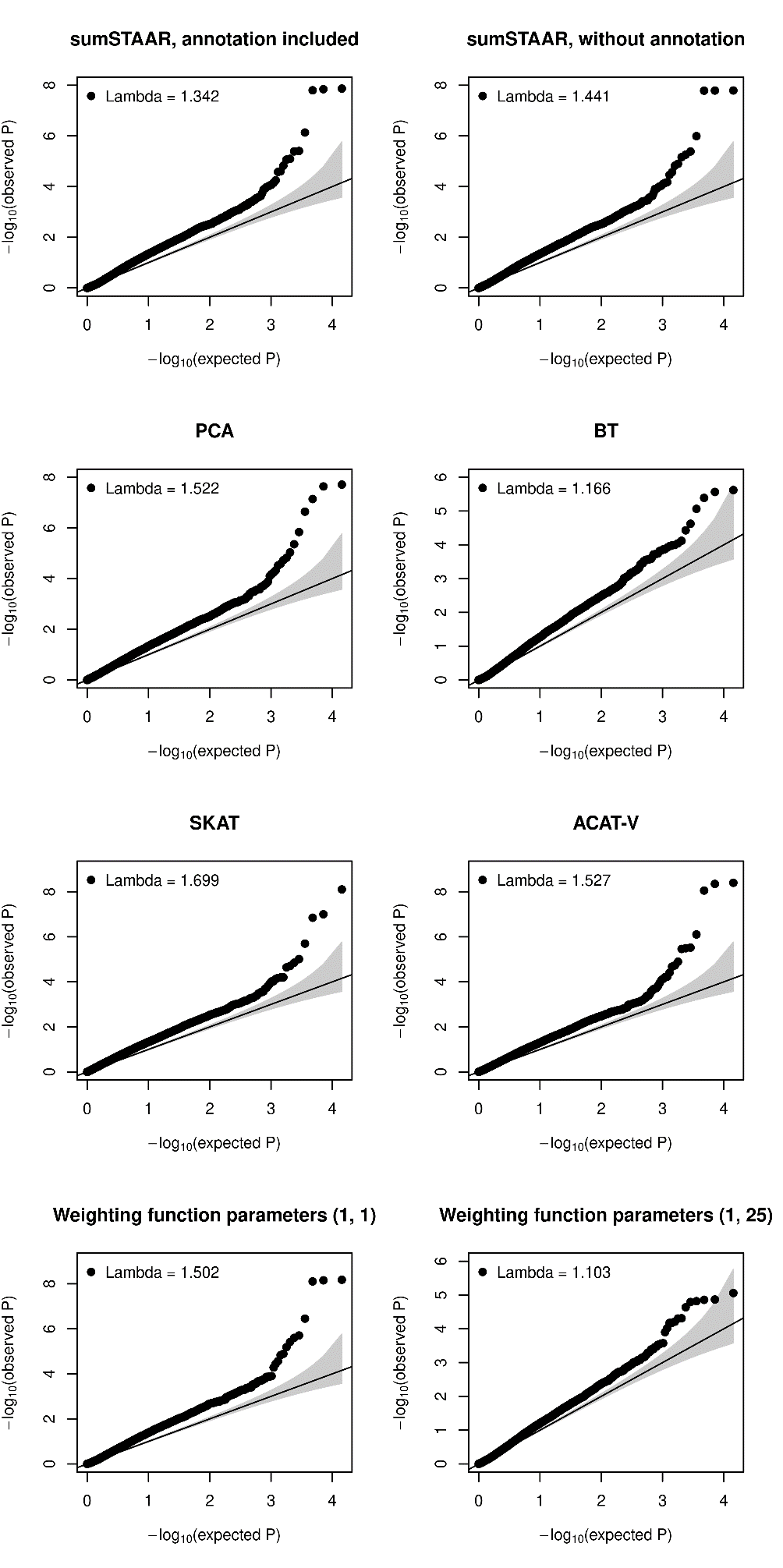


**Fig. S5**. **Q-Q plots for 8 combined tests used in the gene-based analysis of neuroticism**.

The black line is the regression line. The 95% confidence interval is shown in grey.

References

1. Li X, Li Z, Zhou H, Gaynor SM, Liu Y, Chen H, et al. Dynamic incorporation of multiple in silico functional annotations empowers rare variant association analysis of large whole-genome sequencing studies at scale. Nat Genet. 2020;52(9):969-83. doi: 10.1038/s41588-020-0676-4. PubMed PMID: 32839606; PubMed Central PMCID: PMCPMC7483769.
