# Supplementary material for: sumSTAAR: A flexible framework for gene-based association studies using GWAS summary statistics
Source: PLoS Comput Biol. 2022 Jun 2;18(6):e1010172. doi: 10.1371/journal.pcbi.1010172 (PMC9197066; doi:10.1371/journal.pcbi.1010172)
Supplement: Attachment — Submitted filename: Responses_10_03.docx [file pcbi.1010172.s008.docx]

**Response to Reviewers:**

**Reviewer #1**

Inspired by the STAAR framework, the authors extended their previous work in sumFREGAT to sumSTAAR. This paper is concisely written and easy to read. While there are some innovations in combining the two, e.g., new LD matrices, new algorithm for large genes, this reviewer does not find the contributions as significant. In addition, there seemed to be a lack of interest in answering any genetic problem using this new tool. There is no new findings other than the new tool agrees well with previously published ones and the speed is faster. The paper may strengthen if the authors can use this new tool to make significant genetic findings.

**We demonstrated the effectiveness of applying additional methods, weighting functions and functional annotations for gene-based association analysis. Using the neuroticism summary statistics obtained for the protein-coding variants, we showed that inclusion of functional annotations from FAVOR v.2 (http://favor.genohub.org/)** **and two weighting functions allowed us to identify a new neuroticism gene, *NARF*. We presented these results in Supplementary Materials and discussed them in the main text (page 9, lines 20-24).**

**Reviewer #2**

The manuscript describes a statistical framework in attempt to extend the STAAR method to analyze summary statistics data instead of individual genotypes data, and to expand its capacity to be able to incorporate additional models beyond what have been included in the STAAR framework. The extension to analyze summary statistics data is practical and will be useful when implemented properly. This reviewer appreciates the authors’ effort but has several major concerns in this study.

1. For gene-based and/or region-based rare variant analysis, the purpose is to analyze rare variants revealed in sequencing data, to increase power by aggregating multiple potentially causal rare variants that are not usually covered by GWAS data. The current study uses GWAS data for such statistical analysis, which is unlikely equally applicable to sequencing data. In order to make it rigorous so that it can be used by the community, it requires extensive simulation and application to real sequencing data, both of which are severely lacking in the current study.

**We demonstrated that sumSTAAR can be successfully applied to the real exome sequencing data, with its resulting p-values being identical to that of STAAR. We therefore showed that, given appropriate SNP-SNP correlations, sumSTAAR is as effective for WES studies as STAAR. We described these results (page 7, lines 20-26, and Supplementary Materials) and discussed the specifics of using sequencing data in gene-based analysis (from page 11, line 11 to page 12, line 5).**

2. For GWAS, many statistical methods are being developed or extended to summary statistics, which is a huge benefit to the research community given the easy accessibility of summery statistics. The adaptation of such strategy for rare variants uncovered by sequencing, however, is not straightforward. One reason is that the estimates of the effect size and standard deviation are not as accurate for rare variants, and another reason is that the LD among rare variants is challenging to estimate from an external reference panel given that rare variants are often subtly different among sub-populations. Both of the two aspects pose great challenges to the summary statistics-based rare variants analysis. The current study does not address the challenges, and it is not clear how these challenges may affect the analysis results. Extensive investigations are required to evaluate the impact of these issues.

**We agree that the association analysis of rare variants is a challenging task in many aspects. The problem of inaccurate estimates exists, and there are several tools that produce unbiased summary statistics for both binary and quantitative traits (e.g. Zhou et al., 2018, Jiang et al., 2021, Chien, 2020). Now we discuss the issue of summary statistics quality (page 11, line 25 -- page 12, line 5).**

**Regarding the LD estimates for extremely rare variants, we show that the sumSTAAR framework is valid at least when SNP-SNP correlations are calculated on the same sample (page 7, lines 20-26, and Supplementary Materials). We also publish LD correlation matrices for UK Biobank exome sequencing data; they can be used in conjunction with UK Biobank summary statistics (page 12, lines 14-15 and Supplementary Materials). The scope of the framework would increase with more researchers publishing LD matrices together with other summary statistics. We added a discussion of this problem (page 11, lines 16-24).**

3. The inclusion of additional models (e.g. functional linear models) requires additional evaluation to show the benefits of incorporating the additional models. Without such investigation, it is unclear whether the included models are able to increase power or not.

**High statistical power of FLM and PCA has previously been shown for different simulation scenarios and real data (Fan et al., 2013; Svishcheva et al, 2015; Belonogova et al., 2018; Wang and Abbott, 2008; Belonogova et al., 2021). Now we point this out in the discussion (page 9, lines 10-11). Using the results of our previous published study (Belonogova et al, 2021), we compared the performance of the combination of BT, SKAT, PCA and ACAT-V methods and of the smaller set of BT, SKAT and ACAT-V methods. Only 153 of the 190 genes were identified by the smaller set of methods. We discuss these results and the impact of various methods in gene-based analysis on page 9, lines 15-19.**

4. The separation of the SNPs in long genes is arbitrary. Although theoretically sound, in practice in real examples are needed to show that this is a sensible strategy.

**We tested our strategy using neuroticism data as real data example. Figure 3 shows an accuracy and speed of our strategy compared with original analysis without approximation.**

Overall, as this tool is to be used by the researchers, it is critical that this tool is extensively evaluated to make sure that it delivers rigorous results under various scenarios. This is especially critical for rare variants, as it is well-known that there are unique challenges (e.g. point #1 and #2) and such rigorous evaluations are indispensable.

**We performed two additional analyses using real datasets and demonstrated that our sumSTAAR framework generates reliable results, can be applied to exome sequencing data and provide new biological information. We substantially expanded the Discussion and Supplementary Materials sections.**

**Reviewer #3**

In this paper, the authors proposed sumSTAAR as a gene-based association analysis framework using summary statistics from genome-wide association studies (GWAS). The framework allows users to select candidate methods, weighting functions, and probabilities of genetic vairants being causal as introduced in the original STAAR framework. Overall, the proposed framework and data interpretations are justified.

I do have following questions and concerns regarding the manuscript.

Main comments:

1. My main concern is on the «Analysis of large genes» and «Selecting the threshold» in the Methods section. It is known that the original SKAT method (Wu et al. AJHG 2011) is scalable to perform SNP-set test for variant number of several hundreds to thousands. The authors are expected to demonstrate the advantage of computation for variant number more than 10,000. See FastSKAT (Lumley et al. Gen. Epi. 2018) paper as an example.

**We tested our algorithm for large genes using GWAS summary statistics for neuroticism (Fig. 3). The number of variants in the genes was up to 14,000. We extended the discussion of our algorithm and compared it with FastSKAT (page 10, lines 3-22).**

2. Although the proposed sumSTAAR framework uses GWAS summary statistics instead of individual-level data as input. Two main limitations should be discussed clearly by the authors in the revised manuscript:

(1) sumSTAAR could not be directly applied to large-scale sequencing studies, where most of the variants observed are extremely rare variants (e.g. 46% of the observed variants are singletons, see Taliun et al. Nature 2021). The LD matrices for 19,726 genes estimated using the genotypes of 265K participants of UK Biobank would not recover those variants.

**We showed that the sumSTAAR framework can be applied for extremely rare variants at least when SNP-SNP correlations are estimated on the same sample (page 7, lines 20-26). We also discussed the limitations of the rare variants analysis (page 11, line 25 -- page 12, line 5).**

(2) sumSTAAR could not be directly applied to multi-ethnic GWAS studies. As UK Biobank samples are mostly within European ancestry, the use of reference LD matrices calculated from UK Biobank participants may not be generalizable to other ancestries or multi-ethnic studies.

**We agree that matrices we calculated are only suitable for the analysis of European populations. If available, any other matrices calculated, for example, for Asian or African populations, can be used in our framework. We clarified this in the new version of the manuscript (page 8, lines 21-23).**

**To perform multi-ethnic studies, the summary statistics obtained by the meta-analysis can be used together with correlation matrices defined as a weighted combination of matrices for each ethnos as described in Nikpay, M., et al. (2014) Nat Genet 46, 200-204. This approach has been successfully applied by Hindy G, et al. Am J Hum Genet. 2022 Jan 6;109(1):81-96.**

Minor comments:

3. In page 8 line 9, «aggregated Cauchy test, ACAT-O» should be «aggregated Cauchy association test, ACAT». In addition, all the following appearance of «ACAT-O» should be «ACAT», including page 8 line 11; page 9 line 21; page 10, line 2; Figure 1.

**Done**

4. Following #3, in page 13 line 4, suggest changing «… and their ACAT-O-based combinations» to «… and their combinations» for clarity.

**Done**

5. Following #3, in page 13 line 6-8, ACAT-O is a standalone variant-set test that combines the burden test, SKAT, and ACAT-V (Liu et al. AJHG 2019). It is shown that ACAT-O has better statistical power compared to SKAT-O. As such, it would be recommended for the authors to remove the sentence «In addition, … compared with ACAT-O»;.

**Done**

6. In page 14 line 1-3, the conclusion sounds subjective to the broad readership of the journal. Suggest changing to «To  our knowledge, sumSTAAR is a flexible and comprehensive framework that allow researchers to perform state-of-the-art gene based analyses using GWAS summary statistics»;

**Done**

.

7. In page 11 line 23-25, since the authors utilized the STAAR package/tutorial and adapted the scripts based on https://github.com/xihaoli/STAAR/blob/master/docs/STAAR_vignette.html to facilitate the sumSTAAR vs STAAR comparison (https://github.com/nbelon/sumSTAAR-vs-STAAR-comparison/blob/main/sumSTAAR.vs.STAAR.R), it is recommended for the authors to acknowledge the STAAR package authors in the contributor list of the sumFREGAT package (https://cran.r-project.org/web/packages/sumFREGAT/index.html).

**Done**

Have the authors made all data and (if applicable) computational code underlying the findings in their manuscript fully available?

The PLOS Data policy requires authors to make  all data and code underlying the findings described in their manuscript fully available without restriction, with rare exception (please refer to the Data Availability Statement in the manuscript PDF file). The data and code should be provided as part of the manuscript or its supporting information, or deposited to a public repository. For example, in addition to summary statistics, the data points behind means, medians and variance measures should be available. If there are restrictions on publicly sharing data or code &mdash;e.g. participant privacy or use of data from a third party&mdash;those must be specified.

**Reviewer #1**: None

**Reviewer #2**: No: The R code provided is only to generate supplementary FigS2. No computational code or data were provided for the main results including Figure 2 or Figure 3.

**Reviewer #3**: Yes

**We have deposited the computational codes for all generated figures and table at**

**https://github.com/nbelon/sumSTAAR-validation**
