# Supplementary material for: sumSTAAR: A flexible framework for gene-based association studies using GWAS summary statistics
Source: PLoS Comput Biol. 2022 Jun 2;18(6):e1010172. doi: 10.1371/journal.pcbi.1010172 (PMC9197066; doi:10.1371/journal.pcbi.1010172)
Supplement: Attachment — Submitted filename: Responses_11_04.docx [file pcbi.1010172.s009.docx]

Response to Reviewers:

Reviewer #1:

The authors have shown the immediate applicability of the sumSTAAR method using the neuroticism data. However, they were able to identify 1 additional associated gene, out of 190 previously found genes.

**We probably didn't clarify the overall effectiveness of the sumSTAAR framework enough, and this lead to misunderstanding. We demonstrated that using the additional PCA method increased the number of genes by 37, and additional weighting functions and functional annotations yielded one more gene. So, the effectiveness of our framework in this case can be estimated as 25% (38/153 * 100%). We included these statements in the text (page 9, lines 24-27).**

It doesn’t bring confidence to the significance of the method presented. And there is no guarantee that it always increase the number of genes identified.

**We agree that our method, like any other statistical method, cannot guarantee that it always increases the number of genes identified. In simulation studies, an increase in power of a method is usually observed for some scenarios, but not for all.**

In particular, this reviewer agrees with Reviewer #2 that “it is critical that this tool is extensively evaluated to make sure that it delivers rigorous results under various scenarios.” And the emphasis may be on evaluating this method extensively. It is hard to argue this method has been evaluated extensively on various scenarios based on the current revision.

**Our framework represents a combination of several components: methods, procedures, and model assumptions. Most of these components have been extensively studied previously. The utility and reliability of our framework mainly follows from the proven properties of its components, namely:**

1. **In gene-based association analysis, individual genotype and phenotype data can be substituted by the GWAS summary statistics, with results being equivalent (Svishcheva, 2019; Svishcheva et al., 2019).**
2. **The PCA, FLM and SKAT-O methods added to our framework have high statistical power for a wide range of scenarios (Fan et al., 2013; Svishcheva et al, 2015; Belonogova et al., 2018; Wang and Abbott, 2008; Lee et al., 2012).**
3. **Weighting functions and functional annotations increase the power of the gene-based association analysis (Wu et al., 2011; Belonogova et al., 2018; Li et al., 2020).**
4. **The aggregated Cauchy omnibus test allows to analytically combine the results of different gene-based methods (Liu et al., 2019).**
5. **Combining the results of different methods increases the power of the gene-based association analysis (Quick et al., 2020; Li et al., 2020; Posner et al., 2020; Deng et al., 2020; Lou et al., 2020).**

**Our framework allows to integrate these benefits into a single pipeline. We showed its feasibility using the neuroticism summary statistics as an example. Additionally, we tested the framework as applied to the exome sequencing data. We demonstrated the equivalence of results obtained by STAAR and sumSTAAR using real exome sequencing data for CAD. Thus, the results obtained by Li et al. (2020) for STAAR, including simulation studies, can be extrapolated to our framework.**

Minor comment:

In Figure 1, it seems all 6 methods, BT, SKAT, SKAT-O, PCA, FLM, and ACAT-V, take in the exactly the same input. Is that the case? Since this paper emphasis the use of summary statistics, to avoid confusion it would be better for the authors to highlight which method uses summary statistics which doesn’t.

**All 6 methods, BT, SKAT, SKAT-O, PCA, FLM, and ACAT-V, use summary statistics as input. Now we have indicated this in the legend to Figure 1.**

Reviewer #2:

The authors have addressed most of my initial concerns. Here are a few comments that could improve the quality further:

1) QQ-plot (of all p-values) needs to be provided to make sure the type I error is well controlled in each analysis. For example, QQ-plots for FigS4 and for each of the listed 8 combination scenarios as shown in Table S1 (using the p-values from all genes analyzed).

**We have added the QQ-plots to Supplementary Materials (Figs. S5-S6).**

2) The URLs for the SNP-SNP correlation matrices of imputed genotype data of UKBB are not working and need to be fixed.

**Done**

Have the authors made all data and (if applicable) computational code underlying the findings in their manuscript fully available?

The PLOS Data policy requires authors to make all data and code underlying the findings described in their manuscript fully available without restriction, with rare exception (please refer to the Data Availability Statement in the manuscript PDF file). The data and code should be provided as part of the manuscript or its supporting information, or deposited to a public repository. For example, in addition to summary statistics, the data points behind means, medians and variance measures should be available. If there are restrictions on publicly sharing data or code — e.g. participant privacy or use of data from a third party — those must be specified.

Reviewer #1: Yes

Reviewer #2: No:

**We have rechecked the URLs for the SNP-SNP correlation matrices and updated the scripts at https://github.com/nbelon/sumSTAAR-validation with the code for QQ-plots.**
